# Naringenin Ameliorates *Drosophila* ReepA Hereditary Spastic Paraplegia-Linked Phenotypes

**DOI:** 10.3389/fnins.2019.01202

**Published:** 2019-11-19

**Authors:** Barbara Napoli, Sentiljana Gumeni, Alessia Forgiarini, Marianna Fantin, Concetta De Filippis, Elena Panzeri, Chiara Vantaggiato, Genny Orso

**Affiliations:** ^1^Scientific Institute, IRCCS Eugenio Medea, Laboratory of Molecular Biology, Bosisio Parini, Lecco, Italy; ^2^Department of Cell Biology and Biophysics, Faculty of Biology, National and Kapodistrian University of Athens, Athens, Greece; ^3^Department of Pharmaceutical and Pharmacological Sciences, University of Padova, Padova, Italy; ^4^Foundation Institute of Pediatric Research, “Città della Speranza”, Padova, Italy

**Keywords:** endoplasmic reticulum, hereditary spastic paraplegia, naringenin, REEP1, ReepA, UPR

## Abstract

Defects in the endoplasmic reticulum (ER) membrane shaping and interaction with other organelles seem to be a crucial mechanism underlying Hereditary Spastic Paraplegia (HSP) neurodegeneration. REEP1, a transmembrane protein belonging to TB2/HVA22 family, is implicated in SPG31, an autosomal dominant form of HSP, and its interaction with Atlastin/SPG3A and Spastin/SPG4, the other two major HSP linked proteins, has been demonstrated to play a crucial role in modifying ER architecture. In addition, the *Drosophila* ortholog of REEP1, named ReepA, has been found to regulate the response to ER neuronal stress. Herein we investigated the role of ReepA in ER morphology and stress response. ReepA is upregulated under stress conditions and aging. Our data show that ReepA triggers a selective activation of Ire1 and Atf6 branches of Unfolded Protein Response (UPR) and modifies ER morphology. *Drosophila* lacking ReepA showed Atf6 and Ire1 activation, expansion of ER sheet-like structures, locomotor dysfunction and shortened lifespan. Furthermore, we found that naringenin, a flavonoid that possesses strong antioxidant and neuroprotective activity, can rescue the cellular phenotypes, the lifespan and locomotor disability associated with ReepA loss of function. Our data highlight the importance of ER homeostasis in nervous system functionality and HSP neurodegenerative mechanisms, opening new opportunities for HSP treatment.

## Introduction

Receptor expression-enhancing protein 1 (REEP1) is an endoplasmic reticulum (ER) resident protein involved in the SPG31 form of Hereditary Spastic Paraplegia (HSP), a neurodegenerative disorder affecting motoneurons. *In vitro* and *in vivo* studies confer to REEP1 a role in different ER related pathways. REEP1 modifies ER architecture, is implicated in ER stress response and finally has a role in Lipid Droplet (LD) biogenesis (Park et al., 2010; Beetz et al., 2013; Björk et al., 2013; Appocher et al., 2014; Falk et al., 2014; Lim et al., 2015; Renvoisé et al., 2016). The ER is a dynamic organelle characterized by a complex interconnected system of endomembranes, tubules, and sheets. ER sheets are cisternal structures with two closely apposed membranes localized in the perinuclear region, while ER tubules form a reticular network in both perinuclear and peripheral regions (Chen et al., 2013; Goyal and Blackstone, 2013). ER architecture is created and maintained thanks to a continuous process of membrane remodeling, governed by homotypic fusion events, tubulation, and curvature rearrangements. Different families of membrane-shaping proteins are part of this complex regulation (De Craene et al., 2006; Hu et al., 2008; Orso et al., 2009). Some of the major players of ER-shaping are also involved in HSP disease. REEP1 and Reticulon 2 are two of the main ER-shaping proteins involved in HSP (in SPG31 and SPG12 respectively), and due to their particular topology, are supposed to induce high membrane curvature of the ER, a process facilitated by cytoskeleton changes (Park et al., 2010; Beetz et al., 2013; Wang and Rapoport, 2019). On the other hand, Spastin, an ATPase protein mutated in 40% of HSP patients, is involved in the disassembly and remodeling of neuronal microtubules and participates in the maintenance of ER integrity together with REEP1, Atlastin1 and Reticulon 2 (Evans et al., 2006; Sanderson et al., 2006).

ER homeostasis is another important cellular mechanism that participates in ER remodeling. REEP1 is also required in the activation of cellular response in *Drosophila* neuronal ER stress, but the molecular mechanism has not been investigated yet. ER stress is activated by various stimuli, including those of cellular redox regulation or by the accumulation of unfolded proteins in the ER, triggering an evolutionarily conserved pathway, known as unfolded protein response (UPR) (Gumeni et al., 2017). UPR is regulated by three major ER proteins (or ER stress sensors): inositol-requiring protein 1 (IRE1), pancreatic eukaryotic translation initiation factor 2α (eIF-2α) kinase (PERK) and activating transcription factor 6 (ATF6) (Inagi et al., 2014; Gumeni et al., 2017). In physiological conditions, all three effectors bind to the 78 kDa ER chaperone glucose-regulated protein/binding immunoglobulin protein (GRP78/BIP) on their luminal domains (Moreno and Tiffany-Castiglioni, 2015). During ER stress, ATF6 is activated after being cleaved, whereas PERK and IRE1 are activated by self-transphosphorylation (Scheper and Hoozemans, 2015). ATF6 is imported into the nucleus to induce the expression of protein quality control genes, while PERK activates the ubiquitous translation initiation factor eIF2α to upregulate genes involved in redox control, metabolism and folding, and mediates transient inhibition of most protein through ATF4 (Scheper and Hoozemans, 2015). The third UPR sensor, IRE1, alternatively splices inactive X box-binding protein 1 (XBP1) mRNA, producing active spliced XBP1 (sp-XBP1) (Tsaytler et al., 2011), which also controls the expression of several genes encoding for chaperons or involved in endoplasmic reticulum associated protein degradation (ERAD) (Yoshida et al., 2001; Calfon et al., 2002; Credle et al., 2005). UPR is highly conserved among species, also in *Drosophila melanogaster* (Hetz, 2012). Increased protein synthesis requires the expansion of the ER membrane network, thus associating UPR and ER membrane extension (Mandl et al., 2013). However, another study indicates that ER membrane expansion and generation of new ER sheets could act as a stress alleviating response independently of UPR activation, suggesting that ER expansion is an integral part of an effective UPR (Schuck et al., 2009).

Dysregulation of ER homeostasis is also involved in metabolic processes, like gluconeogenesis and lipid synthesis. Lipids, such as fatty acids, are essential components of the cellular membranes and act as signaling molecules. UPR activation results in increased expression of lipogenic markers and promotes LD formation, whereas the loss of LD formation up-regulates UPR response. Also, LDs are believed to work as a temporary depot for proteins destined for degradation (Shyu et al., 2018), thus suggesting a strong correlation between UPR and LD formation. REEP1 has been shown to localize or affect LD turnover in cells and murine models with lipodystrophy (Belzil and Rouleau, 2012; Tan et al., 2014).

In the last years, flavonoids have gained growing interest as therapeutic targets, mostly due to their health-promoting properties, and have been largely investigated on cell survival and stress response pathways. Specifically, several studies highlight the role of flavonoids in the oxidative stress responses and particularly on ER stress (Zhang and Tsao, 2016; Yu et al., 2019; Zeng et al., 2019). Among the different flavonoids, naringenin, a flavanone with estrogenic and anti-estrogenic activities, which is mainly extracted from citrus fruits, displays several biological properties such as antioxidant, antitumoral, antiviral, antibacterial, anti-inflammatory, anti-adipogenic, cardio-protective and neuro-protective activity (Gao et al., 2006; Salehi et al., 2019). In the last 3 years, several works focused on the beneficial effects of naringenin on neuronal development, maintenance and functionality (Hegazy et al., 2016; Wang et al., 2017, 2019). In this study, we used *Drosophila melanogaster* to study REEP1-related HSP disease effects on ER homeostasis. Our data show that the *Drosophila* ortholog ReepA plays an evolutionary conserved function in ER remodeling and the absence of ReepA in flies leads to the activation of two specific branches of UPR pathway, Ire1 and Atf6, and finally induces aging phenotypes. Moreover, naringenin administration restores ER homeostasis, climbing capacity and lifespan defects of ReepA mutant.

## Materials and Methods

### Fly Strains and Materials

Fly stocks were raised on standard medium (yeast 27 g/l, agar 10 g/l, cornmeal 22 g/l, molasses 66 ml/l, nipagin 2.5 g/l, 12.5 ml/l ethanol 96%) and in standard conditions at 25°C and 12:12 h light:dark cycle. The *Drosophila* strains used are shown in Table 1. ReepA^+C591^ was used as a genetic background control. ReepA^–541^ mutant and ReepA^+C591^ flies were maintained on standard food at 25°C and Gal4/UAS crossings were performed at 28°C. All reagents, antibodies and compounds are listed in Table 2.

**TABLE 1:** List of fly stocks used.

**Genotype**	**Source**	**References**	**RRID**
*ReepA^–541^/ReepA^–541^*	O’Kane CJ (Department of Genetics, University of Cambridge, Cambridge, United Kingdom)	Yalçın et al., 2017	RRID:DGGR_123207
*ReepA^+C591^/ReepA^+C591^*	O’Kane CJ (Department of Genetics, University of Cambridge, Cambridge, United Kingdom)	Yalçın et al., 2017	
*UAS ReepA^*E*^ myc*	Generated in our laboratory	See section Materials and Methods	
*UAS H-Reep1*	Generated in our laboratory	See section Materials and Methods	
*UAS HNEU-GFP*	Generated in our laboratory	Kassan et al., 2013 See section Materials and Methods	
*Tubulin-Gal4*	Bloomington		RRID:BDSC_5138

**TABLE 2:** List of reagents and compounds used.

**Reagent**	**Source**	**RRID**	**Catalog number**	**Batch number**
Tunicamycin	Sigma-Aldrich		T7765	078M4017V
Ethanol	Carlo Erba Reagents		308640	V9A122089B
Naringenin	Sigma-Aldrich		W530098	MKBW8466
Hydroxylpropyl-β-cyclodextrin	Sigma-Aldrich		H107	WXBC6699
Sucrose	Applichem		A 1125	R14248
Trizol	Euroclone		EMR507100	062117
Direct-Zol TM RNA MiniPrep kit	Zymo Research, Tustin, CA, United States		R2052	ZRC202785
One Step SYBR Prime Script TM RT-PCR Kit II	Takara-Clontech		RR066A	AIG1932A
GRS FullSample purification kit	Grisp Research Solutions		GK26.0050	7E30114A
Bradford proteins quantification kit	Sigma-Aldrich		B6916	SLBP3810V
Laemmli buffer	Sigma-Aldrich		S3401	SLBC5254V
Tween 20	Sigma-Aldrich		P1379	044K0139
Rabbit anti-phospho eIF2aS1	Abcam	RRID:AB_732117		
Mouse anti-α-tubulin	Sigma-Aldrich	RRID:AB_477593		
Goat anti-rabbit immunoglobulins HRP	DakoCytomation	RRID:AB_2617138		
Rabbit anti-mouse immunoglobulins HRP	DakoCytomation	RRID:AB_2687969		
Triton X-100	Sigma-Aldrich		X100	SLBC2688V

### Generation of Constructs/Transgenic Flies

Full-length H-REEP1 cDNA (606 bp) was previously obtained from HeLa cells RNA extract followed by PCR reaction and cloned in the pUAST plasmid. The ReepA^*E*^ cDNA (867 bp) was obtained from *Drosophila* RNA extract and cloned in the pUAST plasmid. UAS-HNEU-GFP fly line was generated by cloning HNEU-GFP (Kassan et al., 2013) in pUASTattB, and transgenic lines were generated by BestGene Inc service (Chino Hills, CA, United States).

### *Drosophila* Drug Treatments

Tunicamycin (Sigma-Aldrich, MO, United States), previously dissolved in ethanol (Carlo Erba Reagents, Milan, Italy), and naringenin complexed with hydroxypropyl-β-cyclodextrin (Sigma-Aldrich, MO, United States) in a mole ratio of 1:1, were added to standard *Drosophila* food at the final concentration of 0.024 mM and 0.5 mM respectively.

### Starvation Assay

For the starvation assay, third instar larvae were individually selected and placed in Petri plates containing a solution of 20% sucrose (Applichem, Ottoweg, D, Germany) in PBS for 4 h.

### RNA Extraction and Real-Time PCR

The relative expression levels of *Bip, Xbp1 total, Xbp1 spliced, Ldh, Atf4, Gp93, Hrd3, Herp, ReepA* were determined using quantitative real-time PCR. Total RNA was isolated from 5 third instar larvae and was extracted by Trizol reagent (Euroclone, Pero, MI, Italy), and purified using Direct-Zol TM RNA MiniPrep kit according to the manufacturer’s instructions (Zymo Research, Tustin, CA, United States). The concentration and purity of RNA samples were determined using a NanoDrop 2000c spectrophotometer (Thermo Fisher Scientific, Waltham, MA, United States). Real-time PCR (qPCR) was performed on Eco Real-Time PCR System (Illumina Inc, San Diego, CA, United States), using One-Step SYBR^®^ Prime Script TM RT-PCR Kit II (Takara-Clontech, Kusatsu, Japan) as in Castellani et al. (2016). The real-time PCR cycling conditions were: reverse transcription 50°C for 15 min, polymerase activation 95°C for 2 min, followed by 40 cycles of 95°C for 15 s, 60°C for a 1 min; melting curve 95°C for 15 s, 55°C for 15 s and 95°C for 15 s for all target genes. The housekeeping *Rp49* gene was used as an internal control to normalize the data. Relative mRNA expression levels were calculated by the threshold cycle (Ct) value of each PCR product and normalized using a comparative 2^–ΔΔCt^ method (Fantin et al., 2019). Data represented are the result of five independent biological replicates. Each biological sample was loaded in triplicate. The gene-specific primers used are shown in Table 3.

**TABLE 3:** List of primer sequences used in Real Time PCR experiments.

**Gene**	**Primer sequence**
*Bip(Hsc3)*	Fw: 5′-GATTTGGGCACCACGTATTCC-3′ Rv: 5′-GGAGTGATGCGGTTACCCTG-3′
*Xbp1 total*	Fw: 5′-TCTAACCTGGGAGGAGAAAG-3′ Rv: 5′-GTCCAGCTTGTGGTTCTTG-3′
*Xbp1 spliced*	Fw: 5′-CCGAACTGAAGCAGCAACAGC-3′ Rv: 5′-GTATACCCTGCGGCAGATCC-3′
*Atf4*	Fw: 5′-TGCGAGTCTCAGGCG TCTTCATCTT-3′ Rv: 5′-CTGCTCGATGGTTGTAGGAGCTGG-3′
*Ldh*	Fw: 5′-GTGTGACATCCGTGGTCAAG-3′ Rv: 5′-CTACGATCCGTGGCATCTTT-3′
*Gp93*	Fw: 5′-TACCTGAGCTTCATTCGTGGCGTCG-3′ Rv: 5′-GCGGACCAGCTTCTTCTTGATCACC-3′
*Hrd3*	Fw: 5′-GCTGTGAGAAGGCGCTGATCCACTA-3′ Rv: 5′-CCAGCAGTCTTACCCGATGCACAAC-3′
*Herp*	Fw: 5′-CTTACGCGCAGTACATGCAGCAGTT-3′ Rv: 5′-CAGCTGCTCCTGCCACTTGTTGTAC-3′
*ReepA (s,p,j,h,g,e)*	Fw: 5′-ATGATCAGCAGCCTGTTTTC-3′ Rv: 5′-CAGTACATCATTCATTTAACATATTC-3′
*Rp49*	Fw: 5′-AGGCCCAAGATCGTGAAG AA-3′ Rv: 5′-TCGATACCCTTGGGCTTGC-3′

### Lifespan Assay

For lifespan assay, control ReepA^+C591^ and ReepA^–541^ mutant flies were collected after hatching and raised on standard medium or 0.5 mM naringenin enriched food at 25°C in a 12:12 h light-dark cycle. Flies were kept in groups of 20 individuals and were maintained in a *Drosophila* vial. Flies were transferred to a fresh medium every 3 days and death events were scored daily. The experiment was repeated ten times for each genotype.

### Climbing Assay

For climbing assays, 30 flies for each genotype were collected after hatching and were transferred twice a week to tubes containing fresh standard or 0.5 mM naringenin enriched food. Climbing capability was tested six times along the life of these flies (5, 10, 15, 20, 25 and 30 days).

*Drosophilae* were placed in an empty plastic vial with a line drawn 2 cm from the bottom of the tube and allowed to recover from anesthesia for 1 h. Flies were gently tapped to the bottom of the tube and the number of flies above the 2 cm mark at 20 s was recorded as a percentage of flies able to climb the vial. Ten separate and consecutive trials were performed and the results were averaged. The experiment was repeated 10 times for each genotype.

### Western Blotting

For western blots, total proteins were obtained from 10 adult flies using GRS FullSample purification kit (Grisp, Research solutions, Porto, Portugal). The protein levels were quantified using the Bradford proteins quantification kit (Sigma-Aldrich, MO, United States). Each sample was diluted 1:2 with standard 2X Laemmli buffer (Sigma-Aldrich, MO, United States), boiled for 5 min a 95°C and 20–25 mg of proteins were separated on 5% stacking-10% separating SDS polyacrylamide gels. The resolved proteins were transferred electrophoretically to polyvinylidene difluoride (PVDF) membrane as described before (Di Francesco et al., 2015). Membranes were blocked in 10% non-fat dried milk in Tris-Buffer Saline and 0.1% Tween 20 (Sigma-Aldrich, MO, United States) (TBST) as in Floreani et al. (2012) and were incubated using primary antibody rabbit anti-phospho eIF2aS1 (1:1000, ab32157, Abcam, Cambridge, United Kingdom) overnight at 4°C. Mouse anti-α-tubulin (1:2000, T9026, Sigma-Aldrich, St. Louis, MO, United States) was used as loading control. Secondary polyclonal goat anti-rabbit and anti-mouse immunoglobulins HRP (1:2000, DakoCytomation, Glostrup, DK) were used in all cases. The protein bands were detected using the C400 Azure chemiluminescence biosystem (Aurogene, Rome, Italy) and band densities were quantified with ImageJ Fiji 1.52 software. The ratio of the target protein to α-Tubulin was recorded and analyzed. At least three independent biological replicates were used for each genotype and condition.

### Immunohistochemistry

Third instar larvae raised at 25°C or 28°C were harvested, dissected in HL3, fixed in 4% paraformaldehyde for 10 min, washed in PBS containing 0.3% Triton X-100 (Sigma-Aldrich, MO, United States) and mounted on glass slides using Mowiol 4-88 as reported in Mushtaq et al. (2016). For live imaging, larvae were dissected in HL3 and acquired as described previously (D’Amore et al., 2016). All confocal images were acquired using a confocal microscope (Nikon D-ECLIPSE C1) equipped with a Nikon 60x/1.40 oil Plan Apochromat objective using the Nikon EZ-C1 acquisition software as described in Caputi et al. (2017).

### Measure of ER Morphology

For ER morphological analysis, muscles of ten third instar larvae were quantified and analyzed with ImageJ Fiji 1.52 software. All quantitative analyses were performed on muscle 6/7 of abdominal segment A3. Fluorescence intensities were measured on maximum projections of confocal stacks (step size 0.55 μm) taken with a Nikon 60x/1.40 oil Plan Apochromat objective using the Nikon EZ-C1 acquisition software. For each sample, three Region of Interest (ROI) with a range of 800–1000 μm^2^ were analyzed.

To quantify cisterna-like structures, an 8-bit ER image was requested. The image was processed with a manual threshold. Threshold values were 80–255 to eliminate the intensity distortion and 40–255 to preserve the continuity of tubules. Then a binary and open image was created and processed by erosion (subtract pixels) and dilation (add pixels) commands of ImageJ software. In the binary and opened image, tubules were eliminated and cisterna-like structures were isolated. The sheet-like structures were analyzed with Analyze Particle tool and their number and size were quantified (Griffing, 2018).

### Statistical Analysis

Statistical analysis was performed with Microsoft Office Excel 2013 software and Prism version 6.00 for Windows (GraphPad Software, La Jolla, CA, United States). Survival data were analyzed using the log-rank test (Mantel-Cox method).

Significance was calculated using one-way analysis of variance (ANOVA) followed by Dunnett’s or Tukey’s multiple comparison test. Student’s *t*-test for unpaired variables (two-tailed) was used for real-time PCR in young and old flies (Figure 2B) and in climbing assay in Figure 2D. Differences were considered statistically significant at *p* < 0.05(^∗^) and *p* < 0.01(^∗∗^) and *p* < 0.001(^∗∗∗^). Data are presented as means and bars are s.e.m. (standard error of the mean).

## Results

### ReepA Regulates ER Morphology

REEP1 homologs are ER-resident proteins implicated in ER remodeling (Beetz et al., 2013; Zheng et al., 2018). To evaluate the function of *Drosophila* ReepA (firstly named DREEP1 in Appocher et al., 2014) in ER membrane shaping, we analyzed ER structure in *Drosophilae* overexpressing ReepA isoform E (here reported as ReepA^*E*^), which shows the highest homology with human REEP1. REEP1 humanized flies obtained by overexpressing *REEP1* human gene, were also analyzed to identify possible conservation of the REEP1 function (Supplementary Figure 1). Besides the gain-of-function lines, we analyzed the ReepA^–541^ *Drosophila* null mutant (Yalçın et al., 2017) for loss-of-function phenotypes. Two of the most used ER markers in *Drosophila* are Lys-GFP-KDEL (Frescas et al., 2006) and BiP–sfGFP–HDEL (Summerville et al., 2016). Lys-GFP fails to recognize the complexity of ER and discriminate tubules and cisternae (Supplementary Figure 2). On the other hand, BiP–sfGFP–HDEL, defines better the ER structures but it resulted lethal at the pupal stage when expressed with drivers as Elav-Gal4 and Mef2-Gal4 and at the larval stage if expressed with Tubulin-Gal4 driver (Supplementary Figure 3). Therefore, we used a recently published ER marker (HNEU-GFP) to visualize ER structure complexity (Forgiarini et al., 2019). The expression of HNEU-GFP transgene is not lethal, also with strong drivers and at relatively high temperatures (28–29°C), allowing us to distinguish the tubule and sheet-like structure of *Drosophila* muscles in both living and fixed samples (Supplementary Figure 4).

**FIGURE 1:**
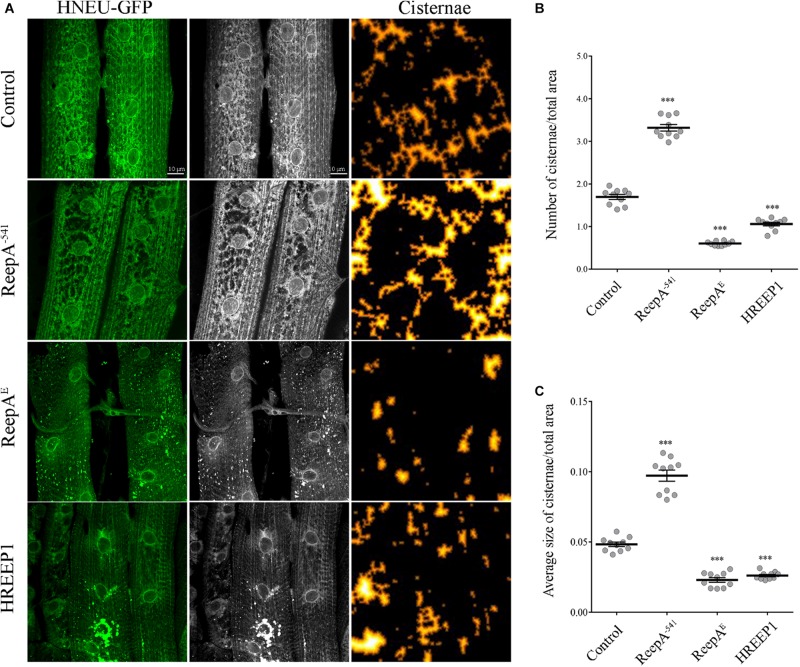
*In vivo* ubiquitous expression of ER-marker HNEU-GFP in *Drosophila melanogaster* ReepA model. **(A)** Representative confocal images of larval muscle 6/7 of abdominal segment A3 of control (*ReepA^+C591^/ReepA^+C591^; Tubulin-Gal4/UAS HNEU-GFP*), ReepA^–541^ mutant (*ReepA^–541^/ReepA^–541^; Tubulin-Gal4/UAS HNEU-GFP*), ReepA^*E*^ (*UAS ReepA^*E*^ myc/*+*; UAS HNEU-GFP, Tubulin-Gal4/*+) and HREEP1 (*UAS HREEP1/UAS HNEU-GFP, Tubulin-Gal4*) larvae, expressing ubiquitously UAS HNEU-GFP. Scale bar = 10 μm. Quantification of cisterna-like structures number **(B)** and size **(C)** of ER-marker HNEU-GFP. Significance was calculated using one-way ANOVA followed by Dunnett’s multiple comparison test. Significance vs. ctr. (^∗∗∗^*P* < 0.001). The bars indicate s.e.m., *n* = 10 larvae.

To quantify the ER defects we ubiquitously expressed UAS HNEU-GFP with Tubulin-Gal4 driver and we followed the protocol described by Griffing (2018). The total area of the ER cisternae was measured along the muscle stacks and quantified as specified in methods section. Both the ubiquitous overexpression of ReepA and HREEP1 and its absence in ReepA^–541^ induced evident morphological alteration of ER architecture, as shown by the HNEU-GFP marker profile (Figures 1A–C). In ReepA^–541^ mutant, the cisterna-like structures were increased and peripheral tubular ER tended to lose its complexity (Figure 1A). On the contrary, the ubiquitous expression of ReepA^*E*^ induced a reduction of cisternae (Figures 1A–C), thus implying a direct role of ReepA on *Drosophila* ER structure modulation. Interestingly, the overexpression of human REEP1 led to a reduction of cisternal structures, a phenotype similar to that of ReepA^*E*^ overexpression, suggesting high conservation of Reep1 function in ER remodeling. Therefore, our data confirmed that *Drosophila* ReepA, similarly to its ortholog REEP1, mediates ER membrane shaping.

### ReepA Is Required During Aging and Stress

**FIGURE 2:**
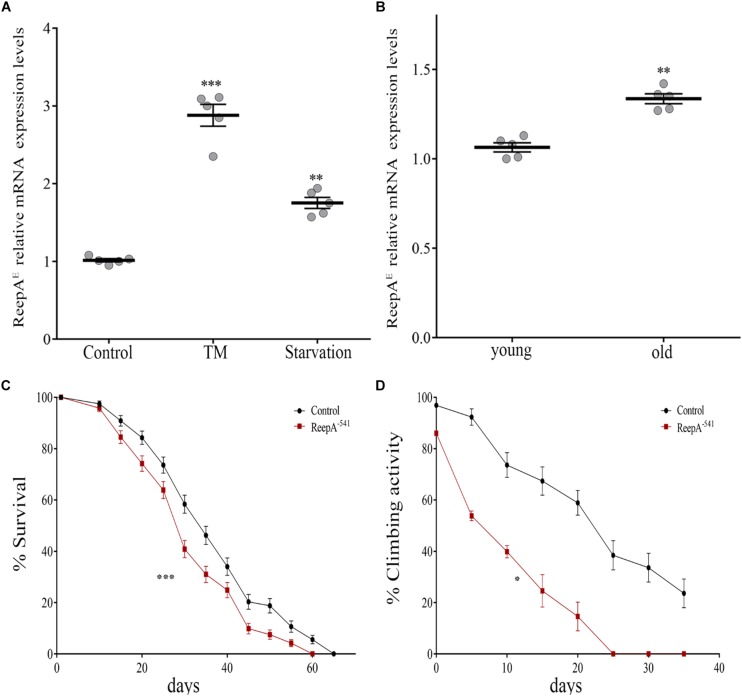
Loss of *ReepA* reduces *Drosophila* longevity and flies’ climbing activity. **(A)** *ReepA*^*E*^ relative gene expression in control (*ReepA^+C591^/ReepA^+C591^*) larvae raised on standard, TM 0.024 mM enriched food, or on starvation conditions. Significance was calculated using one-way ANOVA followed by Dunnett’s multiple comparison test. Significance vs. ctr. (^∗∗^*P* < 0.01; ^∗∗∗^*P* < 0.001). The bars indicate s.e.m., *n* = 5. **(B)** *ReepA*^*E*^ relative gene expression in young (1 day) and old (30 days) control (*ReepA^+C591^/ReepA^+C591^*) flies raised on standard food. Significance was calculated by using two-tailed T-test, ^∗∗^*P* < 0.01. The bars indicate s.e.m., *n* = 5. **(C)** Lifespan in control (*ReepA^+C591^/ReepA^+C591^*) and ReepA^–541^ mutant (*ReepA^–541^/ReepA^–541^*) raised on standard food. Significance was calculated by using the log-rank Mantel-Cox test, *P*-value: ^∗∗∗^*P* < 0.001. The bars indicate s.e.m., *n* = 10 (30 flies for each vial). **(D)** Climbing activity in control (*ReepA^+C591^/ReepA^+C591^*) and ReepA^–541^ mutant (*ReepA^–541^/ReepA^–541^*) raised on standard food and tested at 0, 5, 10, 15, 20, 25, 30, and 35 days. Significance was calculated by using two tailed *T*-test, *P*-value: ^∗^*P* < 0.05. The bars indicate s.e.m., *n* = 10 (20 flies for each vial).

A functional screening performed in *Drosophila melanogaster* demonstrated that ReepA promotes neuronal resistance to ER stress and prevents Tau toxicity (Appocher et al., 2014). Neuronal resistance was tested in adult flies against heat-shock stress and tunicamycin (TM) treatment, identifying ReepA as a new modulator of cellular response to stress. In spite of this, the molecular mechanism remains still unclear. To better understand the role of ReepA under stressors stimuli we first evaluated the transcript expression levels of ReepA^*E*^ after TM chronic treatment or starvation (Appocher et al., 2014; Lindström et al., 2016). Therefore, we compared ReepA^*E*^ transcription levels of stressed wild type larvae versus untreated flies. Our analysis showed that ReepA^*E*^ was upregulated during starvation similarly (but less effective) to TM chronic feeding treatment (Figure 2A). We thus deeply investigated the role of ReepA during aging. We tested the expression level of ReepA^*E*^ in young versus old wild-type adult flies. Adult-aged flies (30 days) expressed higher levels of ReepA^*E*^ compared to 1-day flies (Figure 2B), suggesting an upregulation of ReepA during the adult lifespan. Furthermore, we found that ReepA^–541^ null mutant flies displayed a significant reduction of lifespan (Figure 2C) and climbing ability (Figure 2D), reinforcing the hypothesis that ReepA is required during aging processes. Our data confirmed a crucial role of ReepA in aging and stress conditions.

### ReepA Mutant Flies Triggered a Selective Activation of Atf6 and Ire1 Branches

**FIGURE 3:**
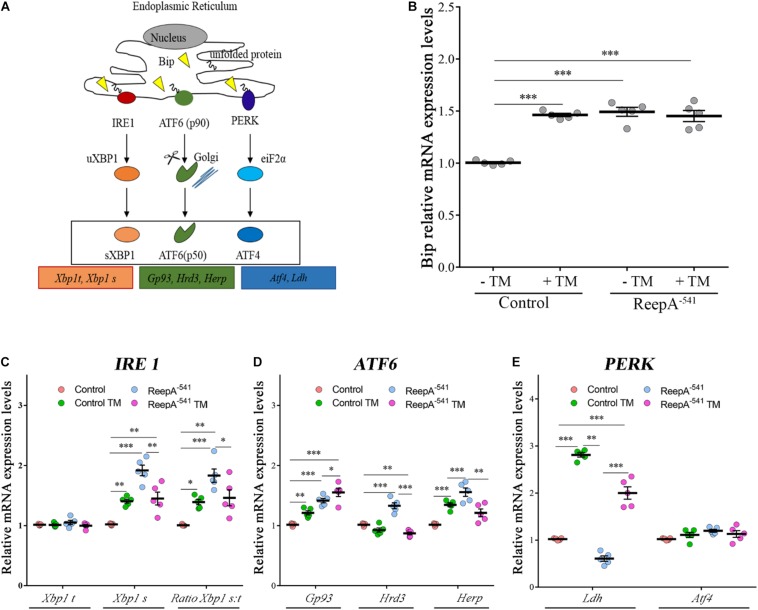
*ReepA* alters the expression levels of genes involved in Ire1/Xbp1, Atf6 and Perk-Atf4 pathways. **(A)** Schematic representation of the three branches of UPR: PERK, IRE1α, and ATF6. Boxes show the genes considered in our analyses. Relative mRNA expression levels of *Bip* **(B)**, *Xbp1 t* and *Xbp1 s* **(C)**; *Gp93, Hrd3* and *Herp* **(D)**; *Ldh* and *Atf4* **(E)** in control (*ReepA^+C591^/ReepA^+C591^*) and ReepA^–541^ mutant (*ReepA^–541^/ReepA^–541^*) larvae raised on standard food or TM 0.024 mM enriched medium. Significance was calculated by using one-way ANOVA with Tukey’s *post hoc* test. ^∗^*P* < 0.05; ^∗∗^*P* < 0.01; ^∗∗∗^*P* < 0.001. The bars indicate s.e.m., *n* = 5.

To elucidate the molecular mechanism underlying ReepA function in cellular stress we tested the effects of the loss of ReepA on UPR response, one of the main cellular signaling activated in case of ER stress (Figure 3A) (Rutkowski and Kaufman, 2007; Mandl et al., 2013). We thus measured the activation of the three main branches of UPR signaling (Perk, Ire1 and Atf6) in *Drosophila* under normal or stress conditions induced by TM administration.

**FIGURE 4:**
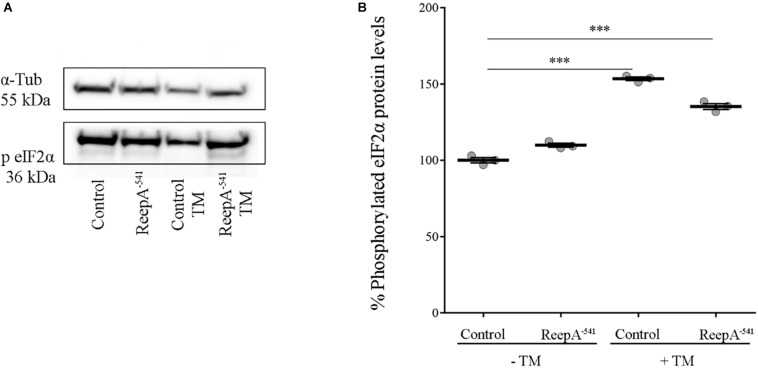
Phosphorylated eIF2α protein expression in ReepA^–541^ mutant and control adults. **(A)** Western blots showing the phosphorylated eIF2α and α-Tubulin (used as the loading control) protein levels in control (*ReepA^+C591^/ReepA^+C591^*) and ReepA^–541^ mutant (*ReepA^–541^/ReepA^–541^*) raised on standard and TM 0.024 mM enriched food. **(B)** eIF2α western blot band quantification. Phosphorylated eIF2α levels were quantified, normalized on α-Tubulin levels and expressed as percent increase versus control. Significance was calculated by using one way ANOVA test with Tukey’s *post hoc* test ^∗∗∗^*P* < 0.001. The bars indicate s.e.m., *n* = 3.

A qRT-PCR analysis on control and ReepA^–541^ third instar larvae, grown on standard or TM enriched food, was performed. Our data showed that the expression of *Hsc3*, the homolog of Bip/GPR94 chaperon, was significantly increased in control larvae treated with TM (Figure 3B), in agreement with previous *in vitro* studies (Lindström et al., 2016). Intriguingly, untreated ReepA^–541^ mutant larvae showed an increase of 1.5 fold in *Bip* transcript levels that remained high even after TM treatment (Figure 3B). The activation of the IRE1 pathway was quantified by measuring the levels of the unspliced [*Xbp1 total (Xbp1 t*)] and IRE1-dependent spliced forms of *Xbp1* [*Xbp1 spliced* (*Xbp1 s*)]. Our analysis showed that the levels of spliced *Xbp1* mRNA were significantly increased in ReepA^–541^ mutant and in control larvae exposed to chronic TM feeding (Figure 3C), supporting the activation of the IRE1 pathway in absence of ReepA. Similar data were obtained by analyzing the Atf6 pathway. Specifically, we found that the Atf6 targets genes *Gp93*, *Herp* and *Hrd3* were upregulated in ReepA^–541^ mutant, mimicking again the response of control larvae treated with TM (Figure 3D). Finally, we evaluated the activation of the Perk pathway by quantifying the expression levels of *Atf4*, but no statistically significant changes were observed in ReepA^–541^ mutant nor in TM treated larvae (Figure 3E). We then considered the expression levels of *Lactate Dehydrogenase* (*Ldh*), a marker of ER stress activation in *Drosophila*, mediated by Atf4 (Lee et al., 2015). Our results showed that *Ldh* expression was strongly increased in control and ReepA^–541^ mutant larvae fed with TM (3 and 2.5 folds as compared to untreated control, respectively), but was decreased in ReepA^–541^ mutant untreated larvae (Figure 3E), suggesting that loss of ReepA does not trigger Perk activation. We further confirmed our findings by measuring the phosphorylated eIF2α protein levels, which were upregulated in TM chronic feeding flies but not in the ReepA loss–of-function background (Figures 4A,B). Thus, our results demonstrate a selective role of ReepA in both Atf6 and Ire1 pathway regulation.

### Defects of ReepA^–541^ Mutant Are Rescued by Naringenin Administration

Naringenin (4′,5,7-trihydroxy flavanone) is a flavonoid that exerts antioxidant activity, triggering the antioxidant system, and it is demonstrated to protect cells from ER stress (Panche et al., 2016; Tang et al., 2017; Salehi et al., 2019). Moreover, like other natural compounds, naringenin has gained attention in the last year as a phytochemical with neuroprotective effects. Several studies described its effects and its ability to inhibit neuro-apoptosis, ameliorate cognitive impairment in different rodent models of neurodegenerative disorders, reduce oxidative stress improving mitochondrial dysfunction in neurons, support dopaminergic neurons and protect from neuroinflammation (Khan et al., 2012; Hsu et al., 2014; Manchope et al., 2017; Wang et al., 2017, 2019; Akang et al., 2019).

One of the stumbling blocks when naringenin or other flavonoids were administrated, is the relatively low bioavailability that could be ameliorated by the complexation with hydroxylpropyl-β-cyclodextrin as described by different groups (Shulman et al., 2011).

**FIGURE 5:**
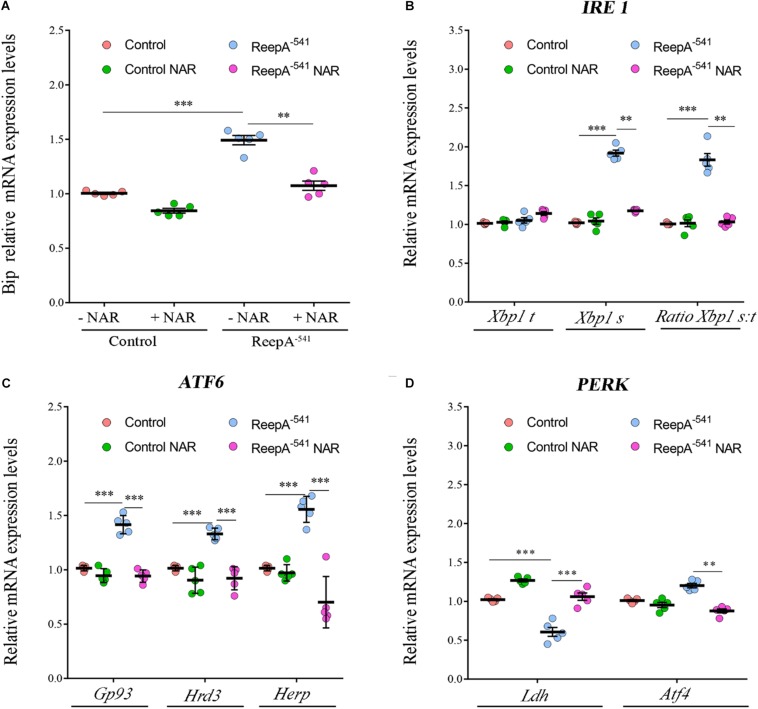
Naringenin restores the expression levels of the genes involved in Ire1/Xbp1, Atf6 and Perk-Atf4 pathways in ReepA^–541^ mutant. Relative mRNA levels of *Bip* **(A)**, *Xbp1 t* and *Xbp1 s* **(B),** *Gp93, Hrd3*, and *Herp* **(C),** *Ldh* and *Atf4* **(D)** in control (*ReepA^+C591^/ReepA^+C591^*) and ReepA^–541^ mutant (*ReepA^–541^/ReepA^–541^*) larvae raised on NAR 0.5 mM enriched or standard food. Significance was calculated by using one-way ANOVA with Tukey’s *post hoc* test. ^∗∗^*P* < 0.01; ^∗∗∗^*P* < 0.001. The bars indicate s.e.m., *n* = 5.

**FIGURE 6:**
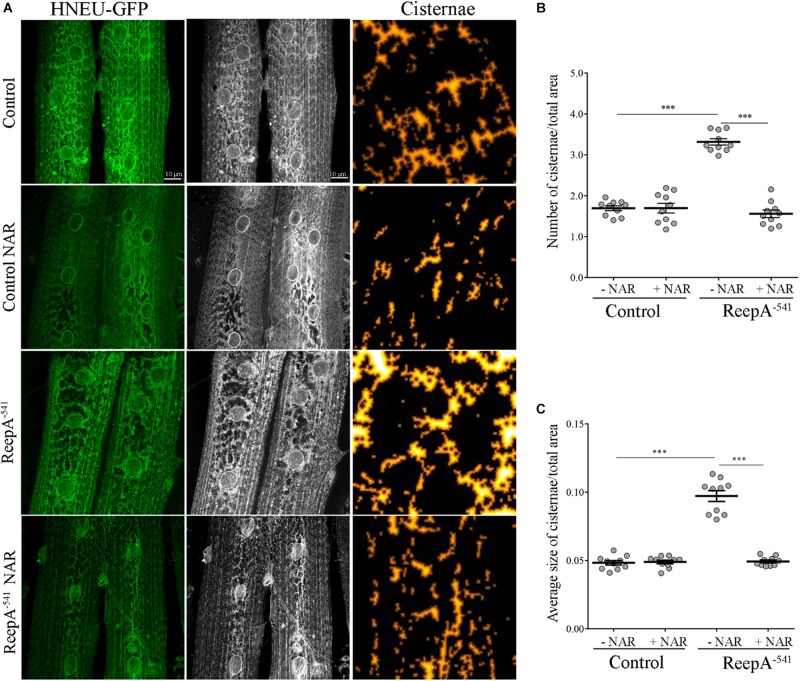
Naringenin rescues ReepA^–541^ mutant ER morphology defects. **(A)** Representative confocal images of muscle 6/7 of abdominal segment A3 of control (*ReepA^+C591^/ReepA^+C591^; Tubulin-Gal4/UAS HNEU-GFP*) and ReepA^–541^ mutant (*ReepA^–541^/ReepA^–541^; Tubulin-Gal4/UAS HNEU-GFP*) third instar larvae expressing ubiquitously UAS HNEU-GFP raised on standard or NAR 0.5 mM enriched food. Scale bar = 10 μm. Quantification of cisterna-like structures number **(B)** and size **(C)** of ER- marker HNEU-GFP. Significance was calculated by using one-way ANOVA with Tukey’s *post hoc* test. ^∗∗∗^*P* < 0.001. The bars indicate s.e.m., *n* = 10 larvae.

**FIGURE 7:**
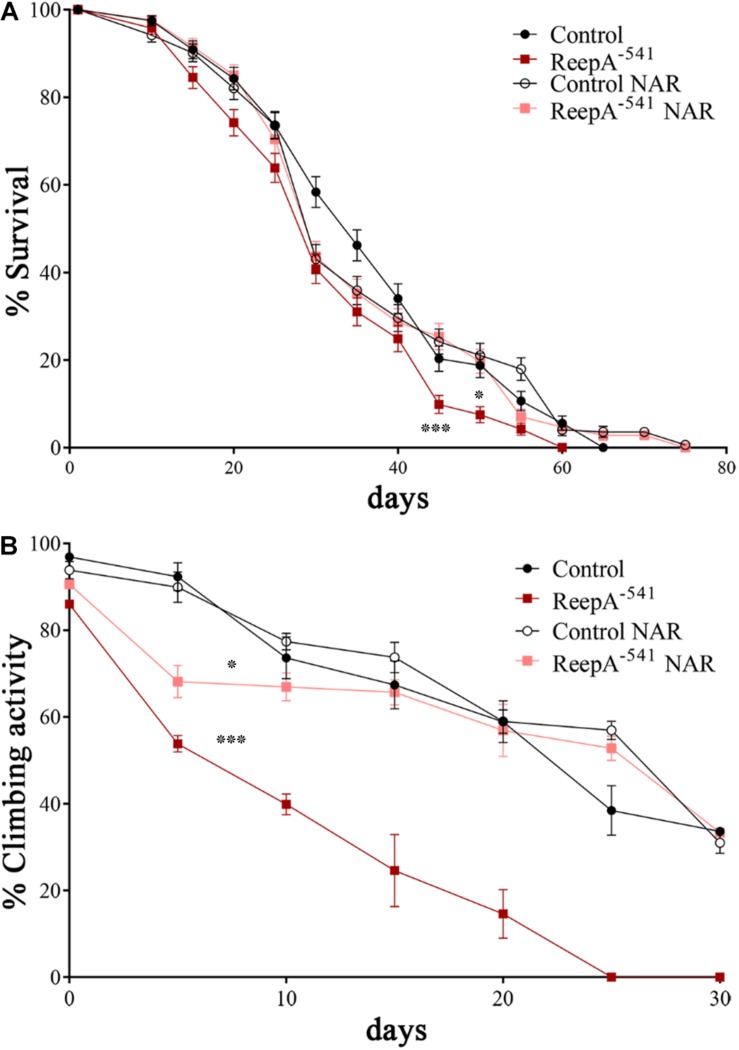
Naringenin ameliorates ReepA^–541^ flies’ lifespan and climbing activity. **(A)** Lifespan in control (*ReepA^+C591^/ReepA^+C591^*) and ReepA^–541^ mutant (*ReepA^–541^/ReepA^–541^*) raised on NAR 0.5 mM enriched or standard food. Significance was calculated using log-rank Mantel–Cox test. *P*-value: ^∗^*P* < 0.05 (ReepA^–541^ mutant NAR vs. ReepA^–541^ mutant), *P*-value: ^∗∗∗^*P* < 0.001 (ReepA^–541^ mutant vs. control), *n* = 10 (30 flies for each vial). **(B)** Climbing activity in control (*ReepA^+C591^/ReepA^+C591^*) and ReepA^–541^ mutant (*ReepA^–541^/ReepA^–541^*) raised on NAR 0.5 mM enriched or standard food and tested at 0, 5, 10, 15, 20, 25, 30, and 35 days. Significance was calculated using one-way ANOVA with Tukey’s *post hoc* test. *P*-value: ^∗^*P* < 0.05 (ReepA^–541^ mutant NAR vs. ReepA^–541^ mutant), ^∗^*P* < 0.05 (ReepA^–541^ mutant vs. control). The bars indicate s.e.m., *n* = 10 (20 flies for each vial).

Therefore, we focused our experiments on testing the effects of naringenin on ReepA^–541^ mutant-associated phenotypes, administrating the flavonoid at the concentration of 0.5 mM after its complexation with hydroxylpropyl-β-cyclodextrin. This concentration was chosen based on a previous report that claims a beneficial effect of naringenin in the range between 0.2 and 0.6 mM in *Drosophila* (Chattopadhyay et al., 2016). We conducted a pilot test using different concentrations of naringenin, on ReepA^–541^ mutant and quantified the expression levels of *Bip* transcript (Supplementary Figure 5). The minimum concentration with rescue effect, 0.5 mM, was used to analyze the effects of the flavonoid on ReepA-linked phenotypes. Larvae were grown in naringenin and the expression levels of the genes involved in UPR response were analyzed. Administration of naringenin completely rescued *Bip, Xbp1 s, Gp93, Hrd3, Herp*, and *Ldh* expression levels in ReepA^–541^ mutant (Figure 5). Of note, as shown in Figure 5, control larvae treated with the flavonoid displayed a transcription profile of UPR genes similar to that of the untreated control. We also analyzed the effect of naringenin on ER morphology and we found that the treatment was able to significantly restore the increased cisterna-like structures phenotype caused by the loss of ReepA (Figure 6). Analogously to what observed for UPR genes, naringenin did not affect ER morphology of control animals. We finally evaluated if the flavonoid was able to restore the ReepA^–541^ mutant related age and climbing phenotypes (Figure 7). The analysis of the average lifespan revealed a significant positive effect (but not total) of naringenin on ReepA mutant flies (Figure 7A, and Supplementary Figure 6). In this case, naringenin also affects the longevity of control flies. We then investigated the flavonoid effect on the climbing activity. Our data showed that, after naringenin administration, ReepA^–541^ flies almost totally recovered their moving deficits (Figure 7B). Thus, naringenin reverted the cellular (ER morphology and UPR activation), age and locomotor associated phenotypes induced by ReepA loss of function.

Overall, our *in vivo* findings further confirm that ER structure and function are crucial in HSP neurodegeneration. Additionally, our results suggest that naringenin exerts beneficial effects in *Drosophila* ReepA HSP disease model and enlists a new compound to the future pharmacological therapy implementation in HSP disorders.

## Discussion

To date, 56 disease-causing variants in REEP1, have been reported. HSP-associated REEP1 mutations are predominantly truncating mutations that have been proposed to act by a haploinsufficiency loss-of-function mechanism (Züchner et al., 2006; Beetz et al., 2008; Schlang et al., 2008; Goizet et al., 2011; Richard et al., 2017). In this study, we explored the role of ReepA in ER homeostasis by investigating the consequences of its loss. Our data showed a functional link between the absence of ReepA and the corresponding activation of two specific branches of the UPR pathway: Ire1 and Atf6. Moreover, we found a morphological adaptation of ER in loss-of-function ReepA animals characterized by the increase of ER membrane sheet-like structures. In parallel to these intracellular alterations, we reported reduced lifespan and deficits of locomotor activity of the mutant flies. The function of REEP homologs in cellular stress has been previously investigated by different groups: HVA22, the plant homolog gene of REEP1, is required to counteract stressful situations by inhibiting the activation of programed cell death in plants (Chen et al., 2002); *Drosophila* ReepA downregulation enhances Tau aggregates, whereas its overexpression results protective, suggesting that ReepA is required to confer stress resistance against the accumulation of unfolded proteins induced by TM (Appocher et al., 2014); DNA damage in human cells triggers tubular ER extension via the p53-mediated expression of REEP1/2 and EI24, and this facilitates contacts between ER and mitochondria (Zheng et al., 2018). All these reports support the role of REEPs in response to stressors, but the molecular mechanism remains still to be elucidated. In our work, we analyzed the activation of the three main pathways involved in UPR response and showed a peculiar selective induction of Atf6 and Ire1 in the absence of ReepA. Moreover, it has been demonstrated that the activation of Atf6 and Ire1, but not of Perk signaling, increases the synthesis of phosphatidylcholine, a key ER lipid, and induces ER expansion (Chiang et al., 2012; McQuiston and Diehl, 2017). When a constitutively active form of ATF6α or Xbp1 is expressed in cultured cells, the ER appears enlarged and distended. On the contrary, PERK pathway has not been implicated in ER biogenesis. The selective activation of Atf6 and Ire1, but not of Perk, in ReepA^−541^ mutant and the concomitant morphological ER aberrations suggest a specific role of ReepA in controlling ER homeostasis. Modulation of UPR after the disruption of optimal membrane rearrangements has already been reported: in *Drosophila*, downregulation of the ER-shaping protein Rtln1, causes partial loss of tubular ER and a significant increase of the ER stress response in epidermal cells and neurons (O’Sullivan et al., 2012); the expression of *RTN3*, a specific receptor for the degradation of ER tubules, is upregulated by ER stress and its loss is associated to attenuated basal ER stress (Chen et al., 2011; Grumati et al., 2017); the *Arabidopsis* mutants of *RHD3*, an ER-shaping GTPase, which have long unbranched ER tubular structures, lack the ability to invoke UPR interfering with IRE1 function (Lai et al., 2014). Although the mechanism by which these ER-shaping proteins regulate UPR is not clear, a link between tubular ER structure and ER stress exists. Indeed, recent findings indicate that the UPR can be directly activated by altering ER lipid composition (Hapala et al., 2011; Volmer et al., 2013; Halbleib et al., 2017). UPR activation is observed in yeast lacking the enzymes required for the biosynthesis of triacylglycerol and sterol esters, and is thus devoid of LDs. *Reep1^–/–^* mice that present ER sheet expansion showed an impairment of LDs and lipoatrophy, with significantly decreased visceral fat (Park et al., 2010; Beetz et al., 2013; Falk et al., 2014; Lim et al., 2015; Renvoisé et al., 2016).

Furthermore, we found that loss of ReepA resulted in a decline in motor ability and a reduction of the lifespan. A previous report, in which another ReepA mutant was described, showed no aging phenotypes in absence of stress, but a drastic reduction of lifespan was seen under stress conditions (Appocher et al., 2014). The discrepancy in this finding could be due to the different fly models used; however, we were not able to obtain the same mutant line to repeat the experiments and compare the data. In our work, climbing test and lifespan analysis were carried out in parallel in a Spastin *Drosophila* model as indicated (Orso et al., 2005; Julien et al., 2016) to compare the results and the methodology (not shown). Based on the outcome, we feel confident that the experimental conditions and methodology were performed correctly.

Finally, we explored the pharmacological effects of naringenin on ReepA *Drosophila* model. Besides the efficacy of naringenin in pathological models of liver diseases, obesity, and diabetes, an interesting role of naringenin is emerged in the last years relating to neurological disorders. The protective effects of naringenin in cellular and animal Parkinson’s models are demonstrated by its ability to restore dopaminergic function, neuro-inflammation and locomotor deficit (Khan et al., 2012; Hegazy et al., 2016; Wang et al., 2019). Naringenin protects motor neuron against methylglyoxal-induced neurotoxicity *in vitro* (Lo et al., 2017). The relatively recent interest in the molecule, the reduced data on pharmacokinetic and metabolic aspects, as well as the chemical instability of this compound have prevented the development of clinical trials activity, at least for now. The increasing attention to naringenin, in fact, has stimulated the researchers to work on delivering systems (Joshi et al., 2018). In this study, we tested the effects of naringenin after the complexation with β-cyclodextrin, an FDA approved excipient that enhances its solubility and increases the absorption rate, as previously reported (Sangpheak et al., 2015; Salehi et al., 2019).

The phenotypes of ReepA loss-of-function model reported in this work were greatly ameliorated by administration of naringenin that rescues not only the phenotypes at molecular and cellular levels, but also restores the climbing behavior as well as ameliorates flies’ lifespan. Overall, our *in vivo* data strongly support the beneficial effects of the natural compound naringenin and open the way for future studies devoted to pharmacotherapy in HSP. We propose a possible protective role of naringenin in HSP neurodegeneration and, therefore, the testing of this compound in additional HSP models. Naringenin inhibits the ER stress in several pathological models. The mechanism by which naringenin acts is poorly understood but most of the studies support the notion that naringenin influences the cellular antioxidant balance through its own chemical structure and by inducing the cell antioxidant system: activating nuclear factor-erythroid 2-related factor 2 (Nrf2) pathway, upregulating superoxide dismutase, catalase, glutathione peroxidase and glutathione transferase and decreasing the expression of *miR-17-3* (Song et al., 2016; Curti et al., 2017; de Oliveira et al., 2017; Tang et al., 2017; Wang et al., 2017, 2019). Naringenin has also the capacity to accumulate in the membrane hydrophobic core, decreasing membrane fluidity in a concentration-dependent manner, and therefore to reduce the interaction between free radical and lipids, blocking lipid peroxidation (Arora et al., 2000).

In this light, future studies of naringenin targets, mechanisms, dosage, and delivering routes could be beneficial also for other disorders that share metabolic and oxidative stress activation, such as amyotrophic lateral sclerosis, myoclonic epilepsy, ataxia, and muscular dystrophy, for which pharmacological treatments are still not available (Fischer et al., 2007; Crimella et al., 2011; Joardar et al., 2015; Wang, 2016).

## Data Availability Statement

The datasets generated for this study are available on request to the corresponding author.

## Author Contributions

BN, SG, and GO conceived and designed the experiments. BN, SG, AF, MF, CD, EP, and CV performed the experiments. BN, SG, AF, CD, EP, CV, and GO analyzed the data. GO contributed to reagents, materials, and analysis tools. BN, SG, and GO wrote the manuscript.

## Conflict of Interest

The authors declare that the research was conducted in the absence of any commercial or financial relationships that could be construed as a potential conflict of interest.
