# Supplementary material for: Naringenin Ameliorates Drosophila ReepA Hereditary Spastic Paraplegia-Linked Phenotypes
Source: Front Neurosci. 2019 Nov 19;13:1202. doi: 10.3389/fnins.2019.01202 (PMC6877660; doi:10.3389/fnins.2019.01202)
Supplement: Supplementary file 1 [file Data_Sheet_1.PDF]

# *Supplementary Material*

## Supplementary Figure 1

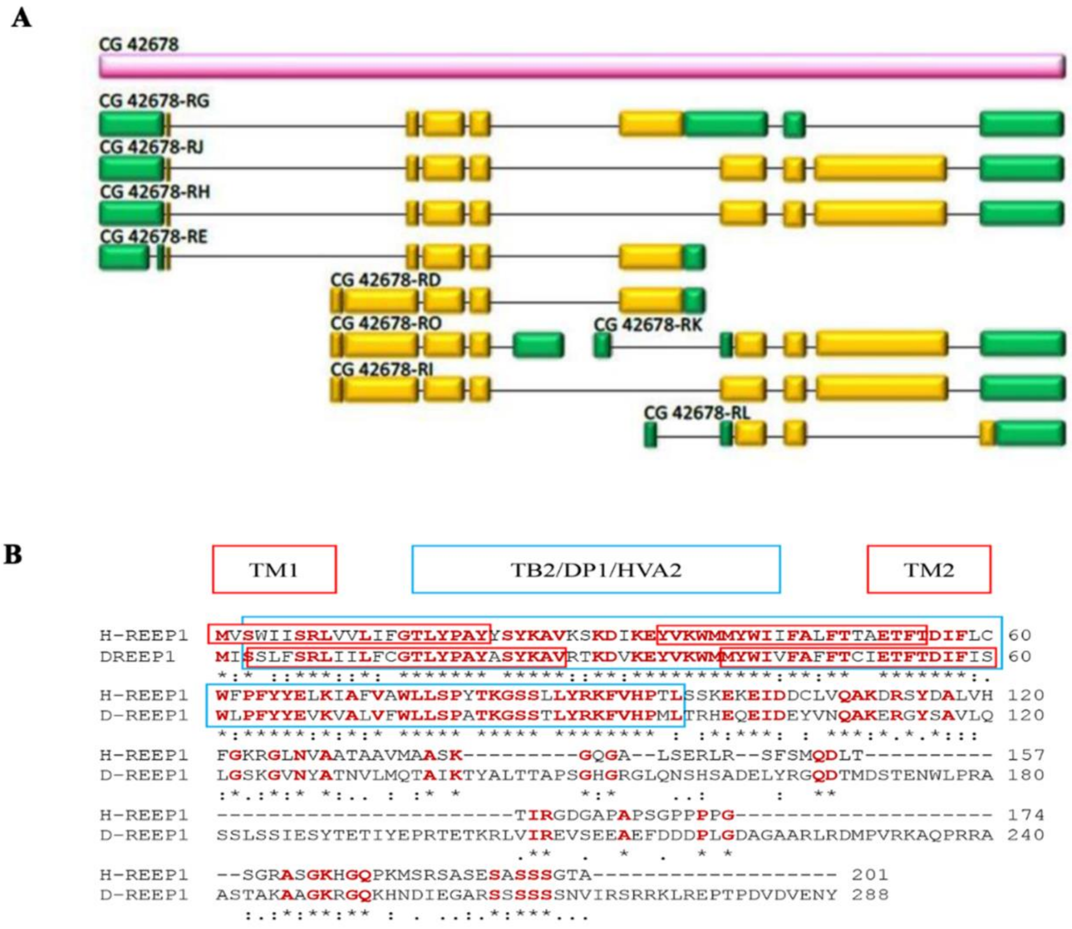


**Supplementary Figure 1.** *Drosophila melanogaster* ReepA transcript map. **(A)** Schematic representation of D-*REEP1*(*ReepA)* gene. The *Drosophila* ortholog of H-*REEP1* gene localizes on the second chromosome and codifies nine transcript isoforms**. (B)** Alignment of human and *Drosophila* REEP1 protein sequence. The conserved amino acids are in red; blu and red boxes are the conserved domain of the protein.

**Supplementary Figure 2**

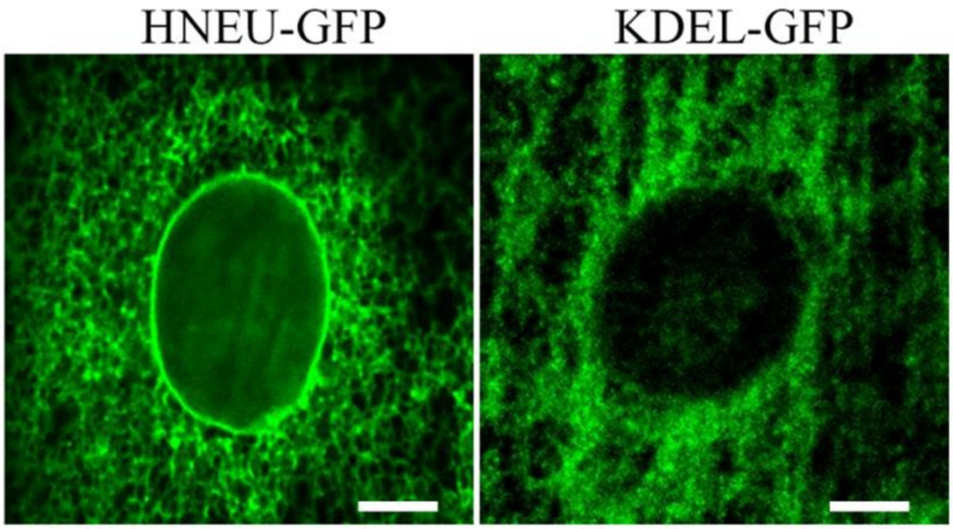


**Supplementary Figure 2.** HNEU-GFP and Lys-GFP-KDEL ER marker fly lines. Representative confocal images of larval muscle 6/7 of abdominal segment A3 of wild type third instar larvae expressing ubiquitously the ER markers HNEU-GFP and Lys-GFP-KDEL (*Tubulin-Gal4/ UAS HNEU-GFP*, *Tubulin-Gal4/ UAS Lys-GFP-KDEL*). Scale bar=10 µm.

**Supplementary Figure 3**

| Driver | UAS-BiP–sfGFP–HDEL | UAS-Lys-GFP-KDEL | UAS-HNEU-GFP |
|---|---|---|---|
| Tubulin-Gal4 | L3 larvae lethal | Not lethal | Not lethal |
| Mef2-Gal4 | Pupae lethal | Not lethal | Not lethal |
| Elav-Gal4 | Pupae lethal | Not lethal | Not lethal |

**Supplementary Figure 3.** Lethality of ER marker fly lines. Stage of lethality of UAS BiP-sfGFP-HDEL, UAS-Lys-GFP-KDEL and UAS HNEU-GFP fly lines overexpressed with different drivers (*Tubulin-Gal4/ UAS BiP-sfGFP-HDEL, Mef2-Gal4/ UAS BiP-sfGFP-HDEL, and Elav-Gal4/ UAS BiP-sfGFP-HDEL; Tubulin-Gal4/ UAS UAS-Lys-GFP-KDEL, Mef2-Gal4/ UAS-Lys-GFP-KDEL, and Elav-Gal4/ UAS-Lys-GFP-KDEL*; *Tubulin-Gal4/ UAS HNEU-GFP, Mef2-Gal4/ UAS HNEU-GFP, and Elav-Gal4/ UASHNEU-GFP*).

**Supplementary Figure 4**

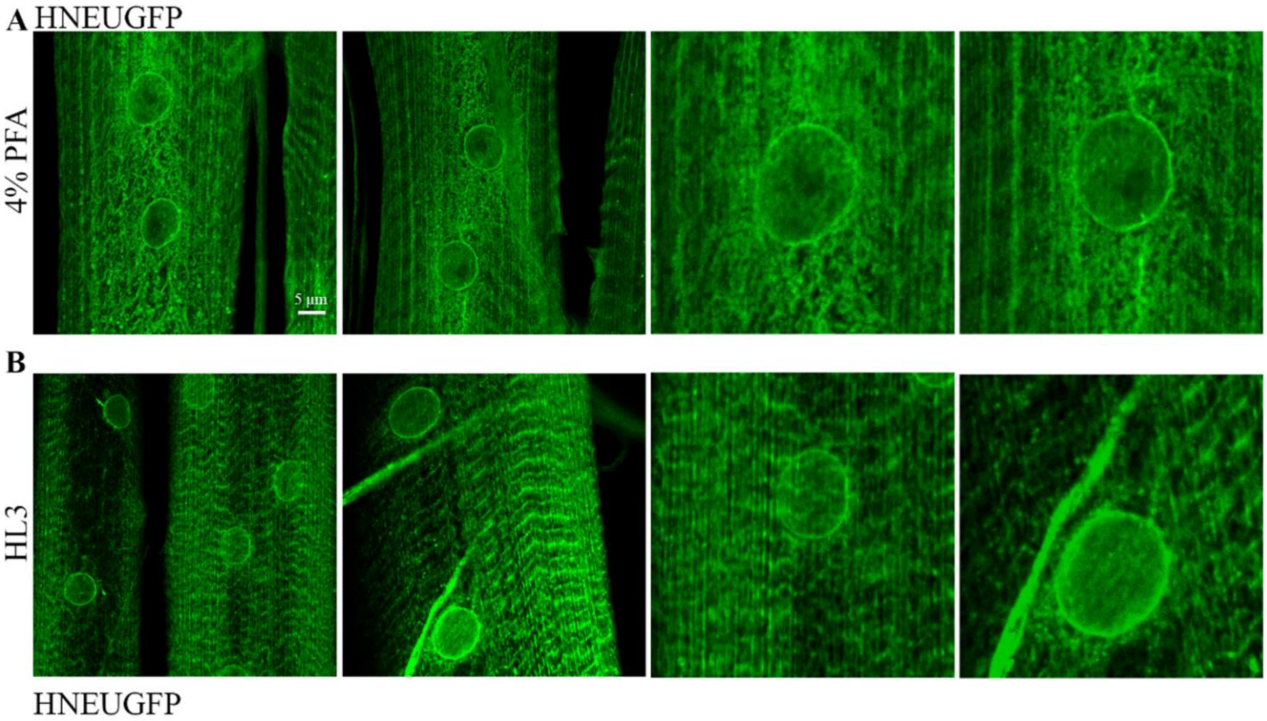


**Supplementary Figure 4.** *In vivo* ubiquitous expression of ER-marker HNEU-GFP in *Drosophila melanogaster* wild type. Representative confocal images of larval muscle 6/7 of abdominal segment A3 of wild type larvae (*Tubulin-Gal4/ UAS HNEU-GFP*) acquired after PFA 4% fixation **(A)** or in live **(B).**

**Supplementary Figure 5**

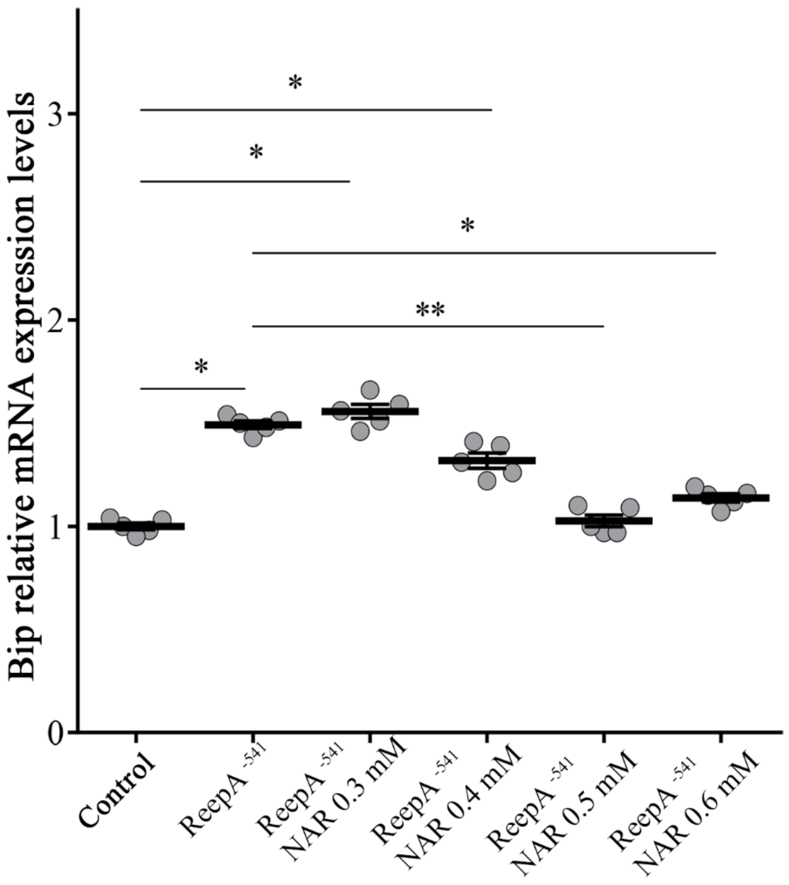


**Supplementary Figure 5.** Real time PCR of *Bip* gene. Relative mRNA expression levels of *Bip* in control (*ReepA*$^{+C591}$/ *ReepA*$^{+C591}$) and ReepA$^{-541}$ mutant (*ReepA*$^{-541}$/ *ReepA*$^{-541}$) larvae raised on standard food or NAR 0.3, 0.4, 0.5 and 0.6 mM enriched medium. Significance was calculated by using one way ANOVA with Tukey's post-hoc test. *P < 0.05; **P <0.01. The bars indicate s.e.m., n=5.

**Supplementary Figure 6**

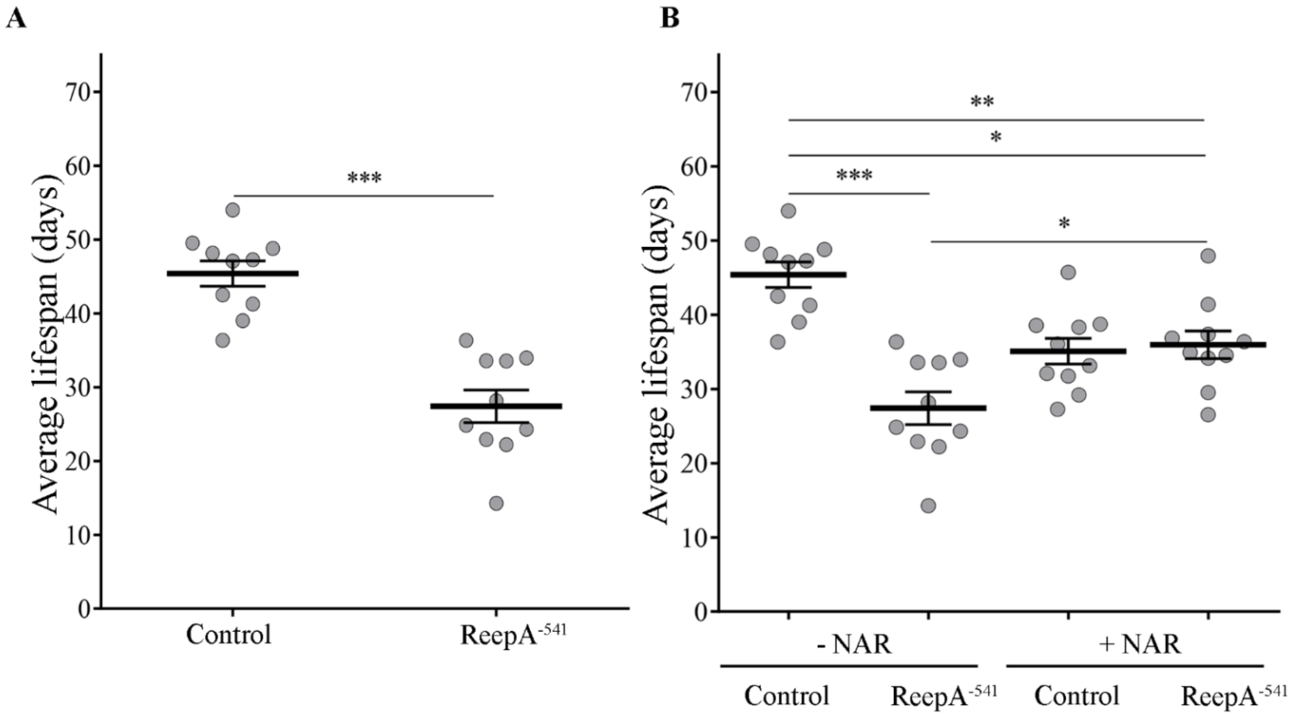


**Supplementary Figure 6.** Average lifespan in control and ReepA$^{-541}$ mutant. Quantification of average lifespan in control (*ReepA$^{+C591}$/ ReepA$^{+C591}$*) and ReepA$^{-541}$ mutant (*ReepA$^{-541}$/ ReepA$^{-541}$*) larvae raised on standard food **(A)** or NAR 0.5 mM enriched medium **(B)**. Significance was calculated by using one way ANOVA with Tukey's post-hoc test. *P < 0.05; **P <0.01, ***P<0.001. The bars indicate s.e.m., n=5.
